# Prevalence and genotype distribution of human papillomavirus (HPV) in Tehran, Iran: a retrospective cross sectional study (2011–2024)

**DOI:** 10.1186/s12985-026-03213-2

**Published:** 2026-06-09

**Authors:** Nader Hashemi, Behnam Mahdavi, Fatemeh Eskandari, Alaleh Alizadeh Halili, Zahra Jamshidi, Maryam Sadat Hosseini

**Affiliations:** 1 Neuroscience Research Center, Biomedical Research Institute, Golestan University of Medical Sciences, Gorgan, Iran; 2 Department of Research and Development, Saeed Pathobiology and Genetic Laboratory, Tehran, Iran; 3 Department of Stem Cell and Regenerative Medicine, National Institute of Genetic Engineering and Biotechnology, Tehran, Iran; 4 Department of Cellular and Molecular Biology, Islamic Azad University, Science and Research Branch (SRBIAU), Tehran, Iran; 5 Preventative Gynecology Research Center, Shahid Beheshti University of Medical Sciences, Tehran, Iran

**Keywords:** Human papilloma virus, HPV prevalence, Sexually transmitted infection (STI), Asymptomatic HPV

## Abstract

**Background:**

Human papillomavirus (HPV) is a highly prevalent sexually transmitted infection in the world, particularly among young adults shortly after they become sexually active. Beyond its commonality, HPV is a significant public health concern due to its carcinogenic potential. Globally, HPV is responsible for a substantial number of cancer cases annually, including the vast majority of cervical cancers, as well as a notable proportion of other anogenital cancers. To convey the urgent need for comprehensive prevention strategies, this study aimed to assess HPV test positivity and genotype distribution among individuals referred for HPV testing in Tehran, Iran, over a 14-year period (2011–2024), providing the foundational data required to guide future national vaccination and screening programs.

**Methods:**

This study examined the demographic characteristics of 6418 individuals (5988 women and 430 men) tested for HPV using data and samples collected from patients visiting a Saeed pathology and genetics laboratory between 2011 and 2024. HPV DNA was extracted from genital samples, (cervical/vaginal specimens for women, and penile/urethral swabs and urine for men) representative of both female and male individuals in Tehran, and analyzed for the presence of HPV. Genotyping was performed by real-time PCR (Cobas 4800 and Sansure G26) and Reverse Hybridization (INNO-LiPA and HPV Direct Flow Chip) methods for the identification of high-risk (HR) and low-risk (LR) HPV genotypes.

**Result:**

Over the 14-year study period, HPV prevalence was assessed using four diagnostic methods (INNO-LIPA, HybriSpot, COBAS X480, and Sansure Kit), reflecting the evolution of available laboratory assays over time yielding positive detection rates of 55.7%, 61.5%, 17.2%, and 52.1%, respectively. The proportion of positive HPV test results varied across age groups, with the highest prevalence observed in the 20–30 year age group. Within the studied cohort, the HPV positivity rate was 40.8% among female patients and 63.3% among the smaller cohort of male patients. The most frequently detected genotypes overall were 6 (12.3%), 16 (7.1%), 52 (3.6%), and 31 (3.3%), in descending order. Notably, 74.4% (517) of patients reported were asymptomic and were referred for routine screening. Among HPV-positive individuals, 69.5% (163) were asymptomatic. Conversely, 23.0% (106) of HPV- negative individuals reported at least one clinical symptom. Positive HPV rates were 56.3% and 24.5% in single and married subjects, respectively.

**Conclusion:**

This study contributes valuable insights into HPV prevalence and genotype distribution in samples collected over a 14-year period in Tehran. The high prevalence of HPV infection, particularly with high-risk Genotypes coupled with the highest prevalence observed in the 20–30 year age group and HPV-16 being common in younger ages, without clinical sign of infection, underscores the necessity for sustained vigilance in monitoring HPV prevalence, early detection through screening, and targeted prevention programs, and comprehensive cervical cancer prevention strategies.

## Introduction

Human papillomavirus, a highly prevalent sexually transmitted infection caused by a double- stranded DNA virus, poses a significant global health challenge [1]. HPV is a ubiquitous pathogens capable of infecting cutaneous and mucosal epithelial tissues, leading to a spectrum of clinical manifestations ranging from benign skin warts to life-threatening malignancies [2]. While commonly associated with genital infections, HPV can affect various anatomical sites, including the hands, feet, and larynx; however, it is most critically associated with infections of the genital region where persistent infection with high-risk (HR) genotypes can progress to intraepithelial neoplasia and invasive cancer [3].

The global burden of HPV-related cervical cancer remains a major public health concern. According to GLOBOCAN 2022 estimates, there were 661,044 new cases and 348,186 deaths from the disease worldwide [4]. There are more than 200 HPV genotypes present, a subset of which are implicated in anogenital cancers [5]. These are broadly categorized as high-risk (HR-HPV) probable high-risk types (HPV 26, 53, and 66) or low-risk (LR-HPV). Approximately 16 h-HPV types, including 16, 18, 31, 33, 35, 39, 45, 51, 52, 56, 58, 59, 68, 73 and 82, are strongly associated with cervical cancer development [2]. Notably, HPV types 16 and 18 account for approximately 70% of all cervical cancer cases globally, while other HR-HPV types contribute to the remaining cases [6]. LR-HPV types, a group which includes but is not limited to HPV 6, 11, 40, 42, 43, 44, 55, 61, 70, 81, and 83, are strongly associated with benign conditions such as genital warts and are frequently detected in benign or mildly abnormal cervical tissue [5, 7]. While LR-HPV infections commonly result in warts, persistent HR-HPV infections are etiologically linked to several cancers, including cervical, anal, and oropharyngeal malignancies [8].

The etiological role of HPV in cervical cancer is further underscored by the near-universal detection of HPV DNA in cervical cancer specimens, contrasting sharply with its infrequent presence in healthy uterine tissue [9]. Although many HPV infections are transient and asymptomatic, clearing spontaneously within two years, persistent infection with HR-HPV genotypes represents a significant oncogenic risk [5]. Factors such as host age, immunity, environmental conditions, and viral characteristics influence the likelihood of disease progression. Individuals with compromised immune systems are particularly vulnerable [10]. Fortunately, cervical cancer is largely preventable through primary prevention strategies, including HPV vaccination, and secondary prevention through screening programs. Clinical trials have demonstrated vaccination efficacy in preventing incident HPV infections and cervical intraepithelial neoplasia [11].

Although cervical cancer incidence is declining in regions with robust screening and vaccination programs, it remains a leading cause of cancer mortality among women worldwide [12]. Understanding HPV epidemiology, including type-specific prevalence and incidence, is essential for developing and implementing effective prevention strategies, such as vaccination and screening programs, to reduce the substantial morbidity and mortality associated with this widespread infection. Given the diverse geographic distribution of HPV genotypes and variations in infection rates across different regions, understanding the local HPV epidemiology is crucial for optimizing vaccination strategies and tailoring cervical cancer prevention and treatment programs [2, 13]. Currently, Iran lacks a centralized national HPV vaccination program, and vaccines (such as the bivalent and quadrivalent options) are primarily accessed opportunistically and paid for out-of-pocket. Furthermore, cervical cancer screening is largely opportunistic rather than a fully organized, population-based national program, typically relying on Pap smear cytology with or without HPV co-testing for women aged 30 and older [14, 15].

The documented association between HPV infection and cervical cancer, especially in women of Asian descent, highlights the importance of ongoing surveillance and targeted interventions [16]. Furthermore, the disproportionate burden of cervical cancer in low- and middle- income countries underscores the urgent need for improved access to HPV vaccination and screening programs globally [17]. Statistical data underscores that cervical cancer ranks as the fourth most common cancer among women worldwide, with a particularly high incidence in low- and middle-income countries [18]. While several studies have investigated HPV prevalence in IRAN the results vary. In Iran, according to a 2018 report by the Institut Català d’Oncologia and the International Agency for Research on Cancer (ICO/IARC), approximately 917 new cervical cancer cases are diagnosed annually. Cervical cancer ranks as the 11th leading cause of cancer among Iranian women [19]. Given the established link between HPV and cervical cancer screening programs employing cytological examinations and HPV typing remain crucial [16].

This study analyzed human papillomavirus testing data from male and female clients at Saeed pathobiology and genetics lab in Tehran, Iran, from 2011 to 2024. Demographic data (age, gender, and marital status), clinical history (past positive results), test methods, and HPV genotype outcomes were examined. This research addresses the knowledge gap regarding HPV prevalence and genotypes in an Iranian population. Specifically, it focuses on both men and women, addressing the paucity of data on HPV prevalence, particularly high-risk (HR) genotypes in Iranian men. Given the continuing public health significance of cervical cancer, this study aims to provide valuable insights into HPV DNA positivity, genotypic identification, associated demographic characteristics and clinical signs, and the distribution of high-risk and low-risk HPV genotypes across different age groups. This data will inform targeted HPV prevention and management strategies within Iran.

## Methods

### Study design and clinical specimens

This retrospective cross-sectional study leveraged data and samples collected from patients visiting Saeed Pathology and Genetic Laboratory 2011 to 2024, to investigate HPV prevalence in the Tehran population. A total of 6,418 genital samples, comprising cervical/vaginal specimens from women and pooled penile/urethral swabs and urine from men were analyzed. The study population was carried out to aged 13 to 75 years, acknowledging the age-related changes in HPV prevalence. The study population also comprised both male and female outpatients referred from various specialists (e.g., gynecologists, urologists) across Tehran province. The clinical indications for testing varied by gender. Women were typically referred for routine cervical cancer screening, or as a follow-up to abnormal Pap smear results. Men were primarily referred for the evaluation of suspected sexually transmitted infections (STIs) or other clinical signs, such as genital warts. This study retrospectively analyzed samples submitted specifically for HPV testing; systematic data on co-testing for other STIs were not collected as part of this study’s scope. For women, cervical specimens were obtained through standard cytology (Thin Prep) as part of routine cervical cancer screening. When HPV testing was clinically indicated outside of routine screening protocols (e.g., in virgins), vaginal swabs were collected by medical personnel. Men, typically referred for STI evaluation or suspected genital lesions, provided two samples: pooled swabs of the urethral meatus and penile/testicular/inguinal skin, and a first-void urine specimen. The urine was collected in sterile, preservative-free containers, with patients explicitly instructed to collect the initial 15–30 mL of the stream to ensure a true first-void sample. Samples were transported under cold chain conditions and stored at 4 °C upon arrival at the laboratory. All participants—whether sampled directly at the laboratory or referred by external specialists—standard laboratory consent forms were signed during the reception and registration process at the laboratory. These forms included explicit permission for the retrospective use of anonymized clinical data for epidemiological research.

### HPV DNA genotyping

HPV DNA genotyping was conducted as part of routine clinical diagnostics at the Molecular Genetics Department of the Saeed laboratory, commencing with sample pre-amplification and DNA extraction. DNA extraction was performed using the High Pure PCR Template Preparation Kit (Roche Diagnostics, USA) according to the manufacturer’s protocol. To ensure the quality and validity of the samples—particularly for vaginal secretions and urine, which may vary in cellularity or viscosity—the concentration and purity of the extracted DNA were evaluated using a NanoDrop™ 2000/2000c Spectrophotometers (Thermo Fisher Scientific, Waltham, MA, USA). Furthermore, test validity for every sample was confirmed through the amplification of the internal control specific to each respective detection assay. This step verified the presence of sufficient exfoliated epithelial cells and the absence of PCR inhibitors. Any samples failing to amplify the internal control were deemed invalid and excluded from the analysis. Because this data was collected retrospectively over an extended timeframe, the genotyping kits used varied depending on routine laboratory upgrades and availability at the time of testing. These included the INNO-LiPA HPV Assay (Innogenetics, Gent, Belgium), the HPV Direct Flow Chip (HybriSpot, Spain), the Cobas X480 HPV test (Roche Molecular Diagnostics), and the Sansure HPV G26 Diagnostic Kit (Hunan, China). Key characteristics of each genotyping kit are summarized in Table 1. In addition, from 2011 to 2019, qualitative PCR with PGMY primers was performed alongside HPV genotyping for initial HPV screening [20].

For each assay, HPV DNA extraction, PCR amplification, hybridization, and result interpretation were performed strictly following the manufacturer’s recommended procedures. Genotypes identified were subsequently categorized into high-risk (HR) and low-risk (LR) groups. HR genotypes included types 16, 18, 26, 31, 33, 35, 39, 45, 51, 52, 53, 56, 58, 59, 66, 68, 73, and 82, while LR genotypes comprised types 6, 11, 40, 42, 43, 44, 54, 55, 61, 62, 67, 69, 70, 71, 72, 81, 83, 84, 89.

**Table 1:** Comparison of HPV genotyping methods, genotype coverage, and limits of detection

Method	Protocol	Target Gene	Genotypes Detection	LOD	Ref
**INNO- LiPA**	Reverse Hybridization	L1	LR: 6, 11, 40, 42, 43, 44, 54, 61, 62, 67, 81, 83, 89 HR: 16, 18, 31, 33, 35, 39, 45, 51, 52, 56, 58, 59, 68 pHR: 26, 53, 66, 70, 73, 82	GS: < 50–437 copies/reaction	[21–23]
**Hybr**i**Spot**	Reverse Hybridization	L1	LR: 6, 11, 40, 43, 44, 54, 55, 61, 62, 67, 69, 70, 71,72, 81, 84 HR: 16, 18, 26, 31, 33, 35, 39, 45, 51, 52, 53, 56, 58, 59, 66, 68, 73, 82	GS: 50–500 GE	[24, 25]
**Cobas X480**	Real-time PCR	L1	HR: 16, 18, OH (31, 33, 35,39, 45, 51, 52,56,58,59,66,68)	GS: 150–2400 copies/ml	[26]
**Sansure G26**	Real-time PCR	N.A	LR: 6, 11, 40, 42, 43, 44, 54, 55, 57, 67 HR: 16, 18, 31, 33, 35, 39, 45, 51, 52, 53, 56, 58, 59, 66, 68, 73	5-500 Copies/reaction	[27]

Details regarding the specific genotypes covered by each assay are based on the manufacturer’s provided specifications. Abbreviations: HPV, Human Papillomavirus; HR, High-Risk; LR, Low-Risk; LOD, Limit of Detection; PCR, Polymerase Chain Reaction. N.A Not Available

### Statistical methods

Specimens were characterized based on reception date, age, gender, test result, positive history status, marital status (single/married), and presenting clinical signs. Data analysis was performed using IBM SPSS Statistics version 26. Descriptive statistics, including frequencies and percentages, were calculated to summarize the sample characteristics. Categorical variables were compared across subgroups using the Chi-square test. The independent samples t-test was employed to compare continuous variables between subgroups. Statistical significance was determined at a p-value of < 0.05 for all analyses.

**Fig. 1:**
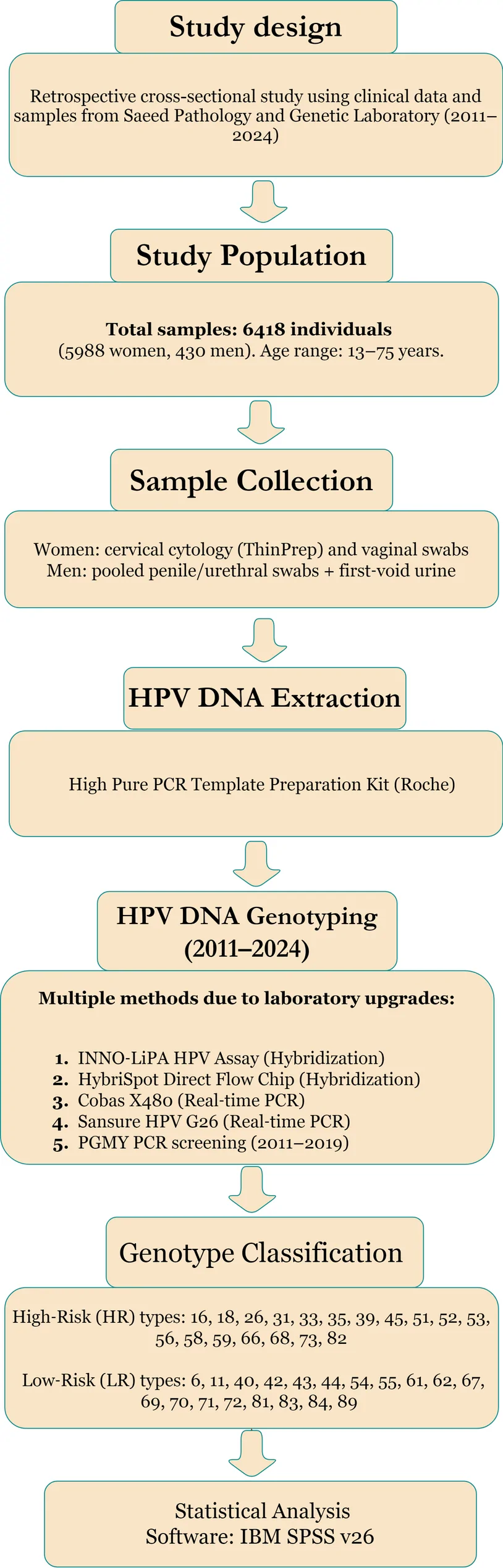
Flowchart of the study design. The schematic outlines the overall framework of the retrospective cross-sectional

To provide a clear overview of the study’s framework, patient selection criteria, and the laboratory workflow for sample processing and HPV genotyping over the 14-year period, a schematic representation is provided in Fig. 1.

Study, detailing the inclusion of male and female outpatients from 2011 to 2024, sample collection, and the progression of HPV genotyping methodologies utilized.

**Table 2:** Demographical and virological characteristics across all participants

	Total	HPV Positive	HPV Negative	*P*-value	OR	LR	HR
Total	6418	2718 (42.3%)	3700 (57.7%)			1593 (24.8%)	1871 (29.2%)
Female	5988 (93.3%)	2446 (40.8%)	3542 (59.2%)	< 0.001	F/M 0.4 (95% CI: 0.33–0.49	1379 (23.0%)	1713 (28.6%)
Male	430 (6.7%)	272 (63.3%)	158 (36.7%)			214 (49.8%)	158 (36.7%)
Mean-Age	35.65	34.19	36.72			33.89	34.24
SD	8.51	8.47	8.38			8.83	8.20
Marital Status	899	299 (33.2%)	600 (66.7%)			135 (15.0%)	241 (26.8%)
Married	652 (72.5%)	160 (24.5%)	492 (75.5%)	< 0.001	S/M 3.958 (CI: 2.908–5.387)	62 (9.5%)	132 (20.2%)
Single	247 (27.5%)	139 (56.3%)	108 (43.7%)			73 (29.6%)	109 (44.1%)

Data are presented as n (%). Percentages represent relative frequencies. The denominator for the percentages in the “HPV Positive” and “HPV Negative” columns is the total number of individuals within that specific column for the respective variable

HPV, Human Papillomavirus; HR, High-Risk; LR, Low-Risk; OR, Odds Ratio; SD, Standard Deviation

## Result

### Demographic characteristics and HPV prevalence

This study examined the demographic characteristics of 6418 individuals (5988 women and 430 men) tested for HPV. The age of participants ranged from 13 to 75 years, with a mean age of 35.6 years (Table 2). Regarding gender distribution, women constituted the vast majority of the study population (93.3%), while men comprised 6.7%. Overall, 42.3% of cases (2718/6418) were HPV-positive. Stratifying by gender revealed that 40.8% of women tested positive for HPV, compared to 63.3% of men. A Chi-square test indicated a significant difference in the proportion of HPV- positive cases between women and men. While the difference between the groups’ percentages was 22.5%, the test of equality yielded a p-value of < 0.001 (Table 2).

**Fig. 2:**
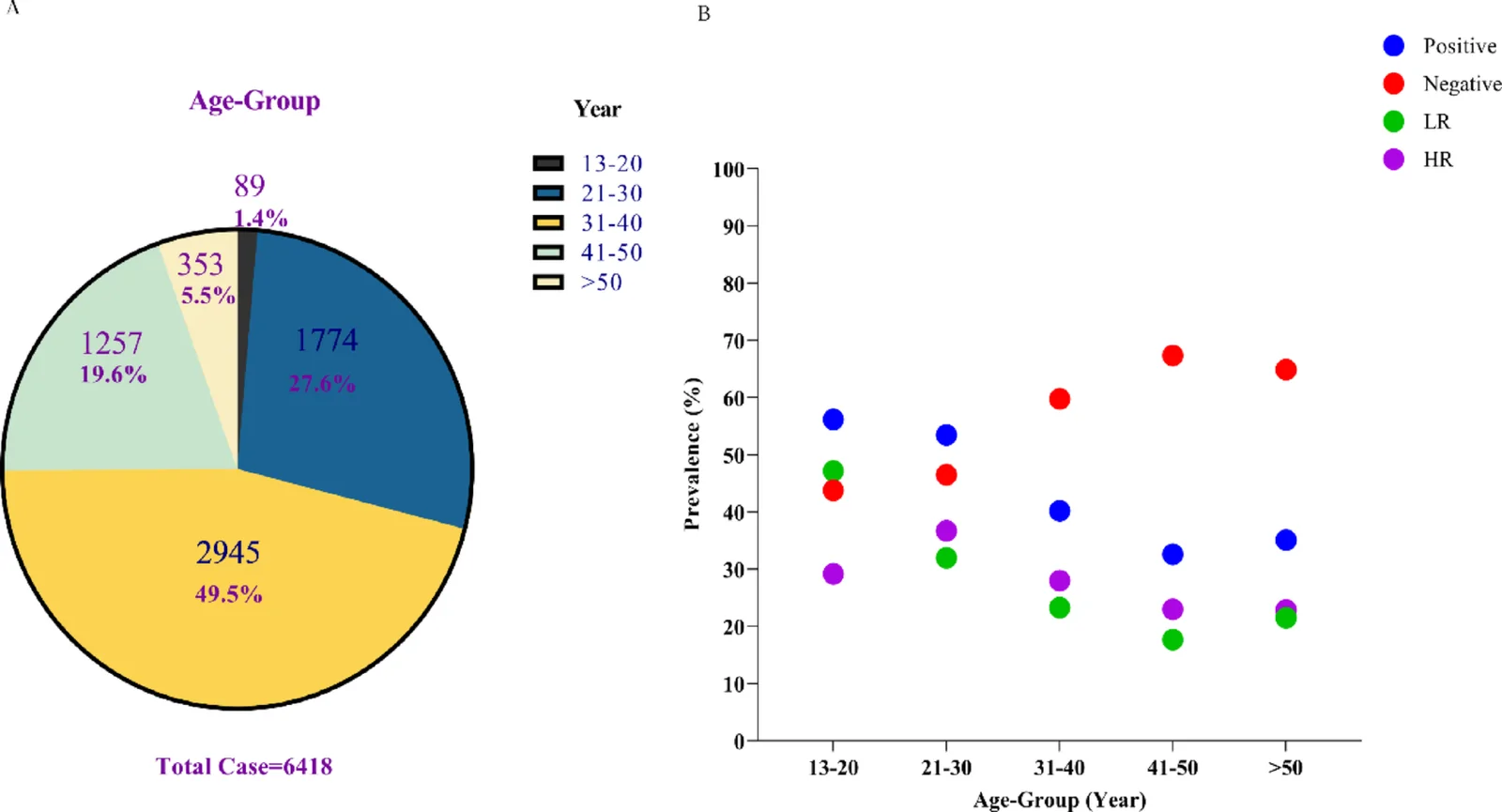
(**A**) Distribution of participants across five age groups, demonstrating the largest proportion in the 31–40 age group. (**B**) Prevalence of positive and negative HPV test results, as well as Low-Risk (LR) and High-Risk (HR) HPV genotypes, across age categories. Highest overall HPV prevalence was observed in individuals under 30 years of age

For subsequent analyses, participants were categorized into five age groups. The distribution of cases across these age groups revealed that the 31–40 age comprised the largest proportion (2945/6418), followed by the 21–30 age group (1774/6418) (Fig. 2a). The proportion of positive HPV test results varied across age groups, with the highest prevalence observed in individuals under 30 years of age (Fig. 2b).

Furthermore, the distribution of both low-risk (LR) and High-Risk (HR) HPV genotypes across different age categories, stratified by gender, indicated the highest prevalence in the 13–20, 21–30 and 31–40 age groups, respectively (Fig. 2b). Analysis using the Mann-Whitney U test demonstrated a significant difference in age between HPV-positive and HPV-negative individuals (U = 4,094,544.5, Z = -12.74, *p* < 0.001). The mean rank age was significantly higher in HPV-negative subjects compared to HPV-positive subjects (Mean Rank 3,461.87 vs. 2,865.95, respectively). This finding suggests an inverse relationship between age and HPV positivity, indicating that older individuals were more likely to test negative for HPV (Table 3).

**Table 3:** Comparison of age distributions between HPV-positive and HPV-negative groups using Mann-Whitney U test

Variable	HPV status	n	Mean rank	Sum of ranks
Age	Negative	3,700	3,461.9	12,808,905
	Positive	2,718	2,866.0	7,789,666
***Test statistic***	**Value**			
*Mann-Whitney U*	4,094,544			
*Wilcoxon W*	7,789,666			
*Z-score*	-12.74			
*p-value*	< 0.001			

### Distribution of HPV genotypes in a referred population

**Fig. 3:**
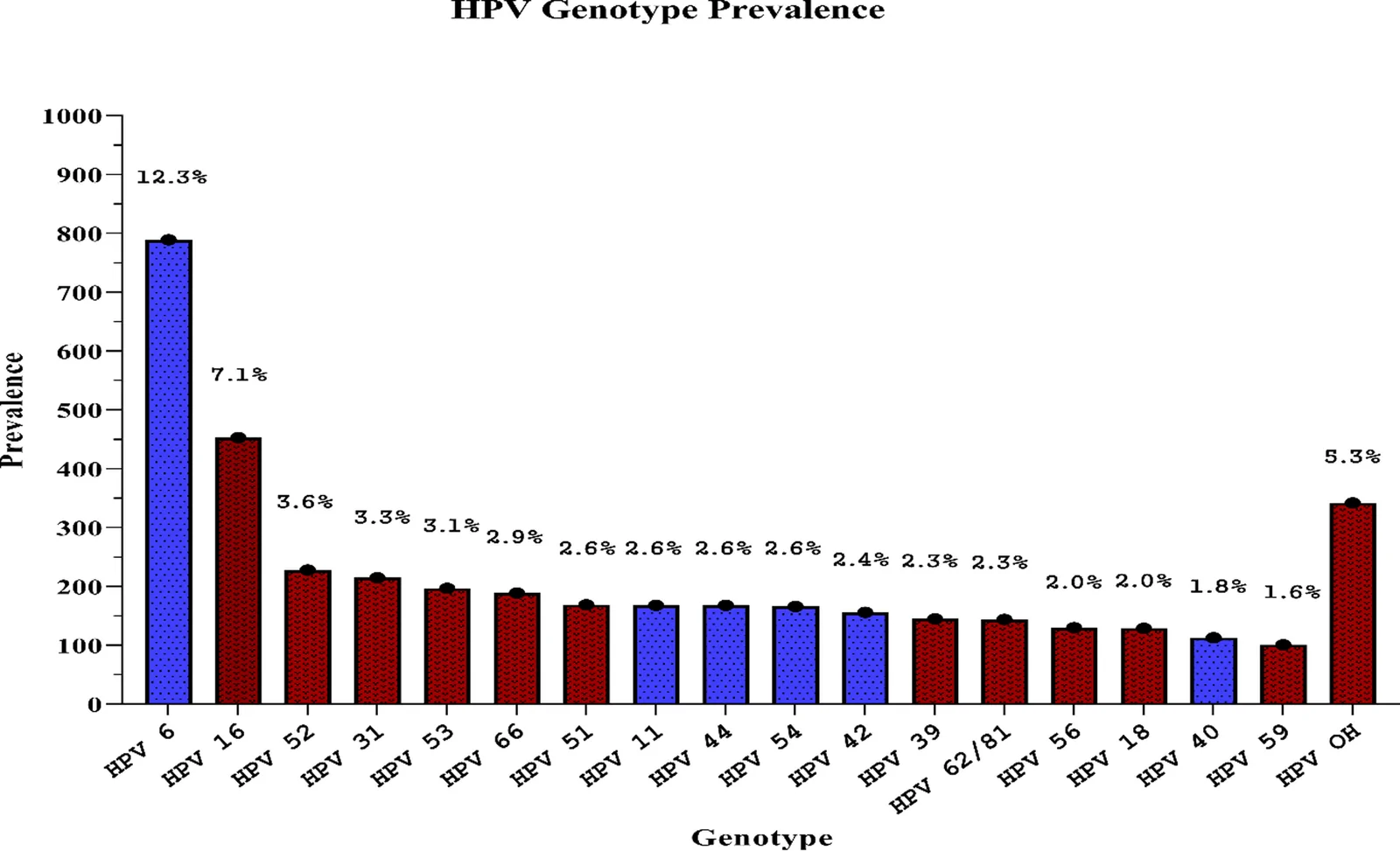
Distribution of HPV genotypes in a population referred for HPV diagnosis. Analysis of all positive cases revealed 34 distinct HPV genotypes. The most frequently detected genotypes for HR was 16, 52 and 31 and for low risk was 6 and 11. The relative frequencies of these genotypes are visually represented

Distribution of HPV genotypes in a population referred for HPV diagnosis indicate a total of 2718 infected cases with 34 distinct HPV genotypes detected. Figure 3 provides a visual representation of the prevalence of the top 18 frequently genotypes identified. The most frequently detected genotypes overall were, in descending order: 6 (12.3%), 16 (7.1%), 52 (3.6%), 31 (3.3%), 53 (3.1%), 66 (2.9%), 51 (2.6%), 11 (2.6%), 44 (2.6%), 54 (2.6%), 42 (2.4%), and 39 (2.3%). Within the HR group, HPV type 16 exhibited the highest occurrence, accounting for 453 cases (7.1%), followed by type 52 with 228 cases (3.6%). Among the LR genotypes, HPV type 6 was the most prevalent, observed in 789 cases (12.3%), with type 11 following at 44 cases (2.6%). Figure 3 illustrates the relative frequencies of these genotypes.

Analysis of the detected genotypes revealed that 1593 (24.8%) were low-risk genotypes, while 1871 (29.2%) were high-risk genotypes (Table 2). Notably, 751 (27.6%) individuals exhibited co-infection with both LR and HR genotypes. Further analysis examined the prevalence of HR and LR genotypes within gender subgroups. In females, 1713 (28.60%) HR genotypes and 1379 (23.0%) LR genotypes were detected. Males exhibited a higher proportion of HR genotypes, with 158 cases (36.7%), compared to LR genotypes, at 214 cases (49.8%) (Table 2). Across both genders, HPV-6 emerged as the most prevalent genotype, occurring in 11.3% of females and 25.6% of males.

**Fig. 4:**
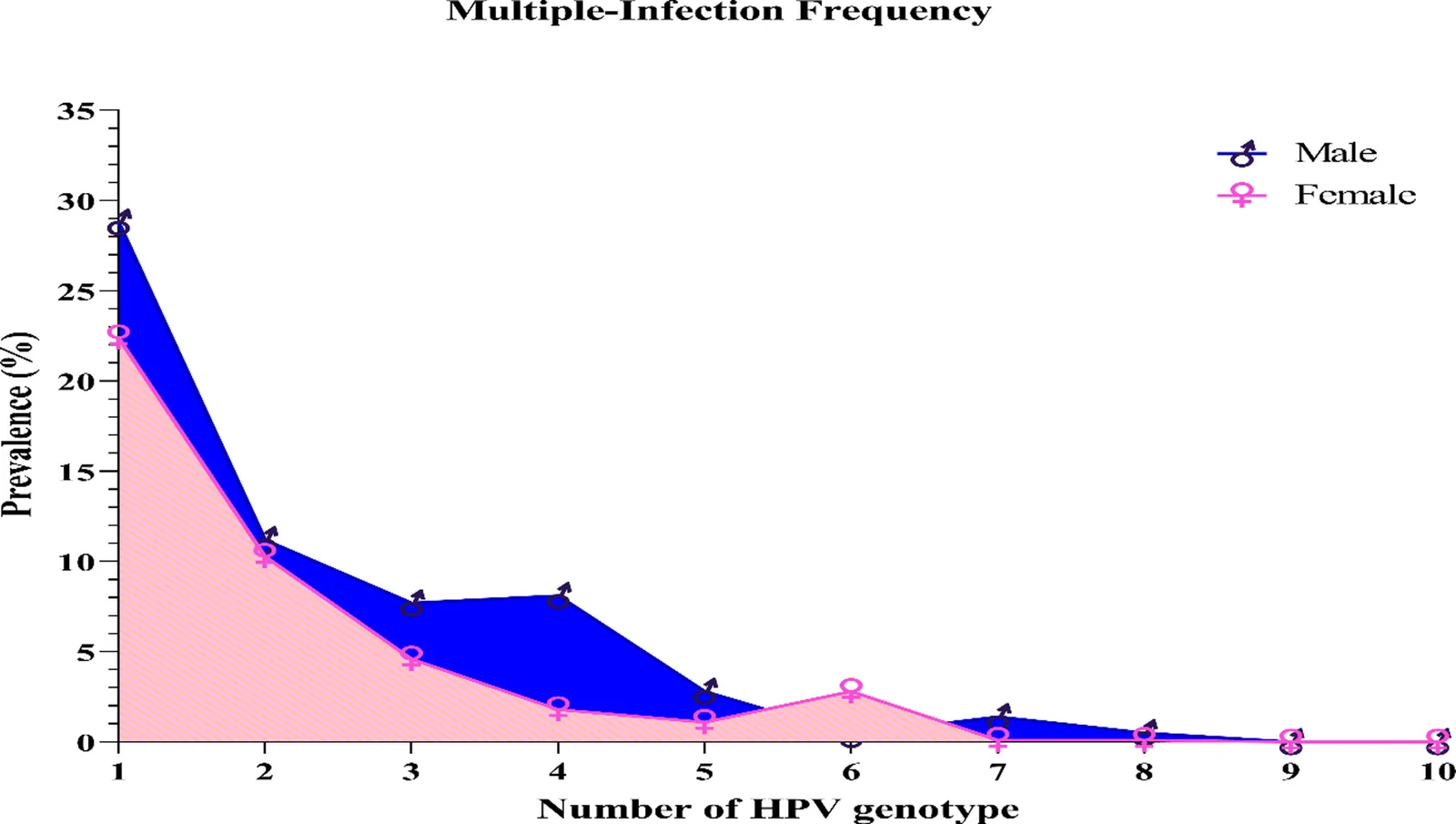
Distribution of single and multiple HPV infections by gender. The graph displays the prevalence of infections involving one, two, three, or more distinct HPV genotypes. Data for female patients are represented by the pink line and shaded area, while data for male patients are represented by the blue line and shaded area

Single infections represented the predominant infection pattern, observed in 1463 cases (22.8%). In cases of multiple infections, double infections were the most frequent manifestation, present in 664 cases (10.3%). The most complex infection observed involved the detection of 10 different HPV types in two individual cases, identified using a comprehensive diagnostic protocol (Fig. 4).

### Analysis of HPV history and symptomatic presentation in patients referred for HPV testing

This study examines the HPV history and clinical presentation of patients referred for HPV testing between 2023 and 2024. Of the 892 patients who completed questionnaires regarding their HPV history, a significant disparity was observed between those with and without prior HPV testing. Within the study cohort, 671 patients reported no prior positive HPV test, while 221 reported a history of a positive HPV result. Among those with a prior positive HPV result, approximately 49.8% tested positive again. Conversely, 50.2% cleared the virus, although the origin of prior positive results from external laboratories introduces a degree of uncertainty (Table 5). Also, in patient with prior history mentioned, the 41–50 age group exhibited the highest HPV clearance rate (Table 4). A limitation of this analysis is the lack of information regarding the timing of initial infection and previous positive test results, precluding a comprehensive assessment of long- term clearance rates.

**Table 4:** Association between age-group and HPV test outcome with prior history

Age- Group	History of Positive Result	Positive cases	Negative cases	Total
	**No**	3	2	5 100.0% 2 100.0% 108 100.0% 42 100.0% 291 100.0% 93 100.0% 201 100.0% 64 100.0% 66 100.0% 20 100.0% 671 100.0% 221 100.0% 892 100.0%
		60.0%	40.0%	
**13–20**				
		2	0	
	**Yes**			
		100.0%	0.0%	
	**No**	38	70	
		35.2%	64.8%	
**21–30**				
		21	21	
	**Yes**			
		50.0%	50.0%	
	**No**	86	205	
		29.6%	70.4%	
**31–40**				
		49	44	
	**Yes**			
		52.7%	47.3%	
	**No**	45	156	
		22.4%	77.6%	
**41–50**				
		26	38	
	**Yes**			
		40.6%	59.4%	
	**No**	16	50	
		24.2%	75.8%	
**+ 50**				
		12	8	
	**Yes**			
		60.0%	40.0%	
	**No**	188	483	
		28.0%	72.0%	
**Total**	**Yes**	110	111	
		49.8%	50.2%	
	Result	298	594	
		33.4%	66.6%	

Data are presented as n (%)

Furthermore, the study investigated the association between clinical symptoms and HPV test results in a subset of 695 patients. Participants were questioned about genital symptoms, including pain, bleeding, abnormal discharge, itching and burning, and the presence of lesions. Overall, 517 (74.4%) of these 695 patients reported no symptoms. When stratified by HPV test results, 69.4% (163 of 235) of all HPV-positive individuals were asymptomatic. Conversely, 23.0% (106 of 460) of all HPV-negative individuals reported at least one clinical symptom (Table 5).

**Table 5:** Clinical signs and history findings in relation to HPV status among female patients

	Total	Condition		Negative	Positive	P-Value	OR	LR	HR
History	892	Yes	221 (24.8%)	111 (50.2%)	110 (49.8%)	<0.001	2.54	58 (26.2%)	80 (36.2%)
		No	671 (75.2%)	483 (72.0%)	188 (28.0%)			76 (11.3%)	161 (24.0%)
Clinical Sign	695	Yes	178 (25.6%)	106 (59.6%)	72 (40.4%)	0.03	1.47	42 (23.6%)	50 (28.1%)
		No	517 (74.4%)	354 (68.5%)	163 (31.5%)			58 (11.2%)	144 (27.9%)
Pain	43 (6.1%)			32 (74.4%)	11 (25.6%)			4(9.3%)	8 (18.3%)
Bleeding	26 (3.7%)			17 (65.4%)	9 (34.6%)			4 (15.3%)	7 (26.9%)
Abnormal Discharge	45 (6.5%)			31 (68.9%)	14 (31.1%)			6 (13.3%)	13 (28.8%)
Itching and Burning	58 (8.4%)			37 (63.8%)	21 (36.2%)			11 (18.9%)	19 (32.7%)
Lesion or Wart	69 (9.9%)			26 (37.7%)	43 (62.3%)			31 (44.9%)	25 (36.2%)
Check Up (No Sign)	517 (74.4%)			354 (68.5%)	163 (31.5%)			58 (11.2%)	144 (27.8%)

This table presents data exclusively for female participants. Male participants were excluded from this specific clinical manifestation analysis due to differences in anatomical and symptomatic presentations. Data are presented as n (%). Denominators for percentages are based on the total number of individuals within the history and symptom category. Symptoms such as pain, discharge, itching, and burning are non-specific and may be indicative of concurrent genital tract infections rather than solely HPV. Statistical significance was evaluated using Pearson’s Chi-square test, with significance defined as *p* < 0.05

HPV, Human Papillomavirus

### Variability in HPV detection rates: methodological considerations and marital status association

This study analyzed HPV detection rates across several diagnostic methods and explored the relationship between marital status and test results. As detailed in the methods section and corresponding Table 1, the protocols, genotype targets, and lower limits of detection varied across the diagnostic methods employed. This heterogeneity complicates direct comparisons of overall HPV prevalence, but offers valuable insights into the performance and limitations of each assay.

**Fig. 5:**
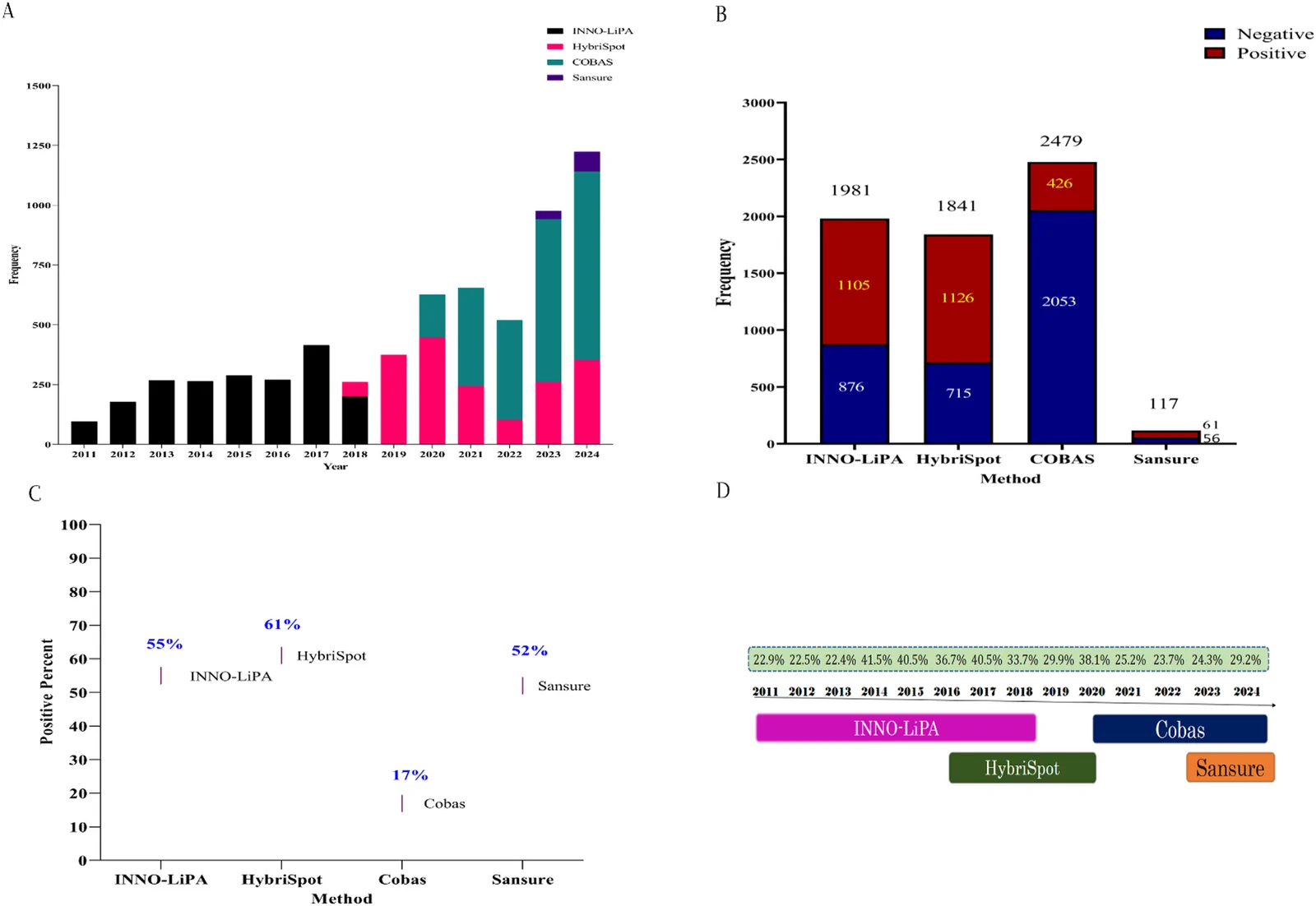
Comparative analysis of HPV detection rates using four diagnostic methods (INNO-LiPA, HybriSpot, Cobas X480, and Sansure G26). (**A**) Annual frequency of tests conducted for each method from 2011 to 2024. (**B**) Total number of positive and negative results obtained by each method. (**C**) Overall positive detection rates for each assay (55%, 61%, 17%, and 52%, respectively). (**D**) Timeline illustrating the period of utilization for each assay alongside the annual overall HPV positivity percentages. Note: The lower detection rate observed with Cobas X480 is attributed to its restricted focus on 14 high-risk genotypes and a higher limit of detection compared to the other assays

Specifically, four diagnostic methods were compared: INNO-LIPA, HybriSpot, COBAS X480, and Sansure HPV G26 Diagnostic Kit. These methods yielded positive detection rates of 55.7%, 61.5%, 17.2%, and 52.1%, respectively (Fig. 5a and b). HybriSpot demonstrated the highest positive detection rate in this comparison. The COBAS X480 exhibited a substantially lower percentage compared to the other methods, likely due to its focus on detecting only 14 high-risk HPV genotypes and its comparatively higher limit of detection. These observed differences in positivity rates may stem from variations in assay sensitivity, the range of HPV genotypes targeted, and potential differences in the underlying patient populations tested.

Further analysis examined the association between marital status and HPV test results within the 2023–2024. Among the 899 patients in the 2023–2024 cohort, 72.5% (*n* = 652) were married and 27.5% (*n* = 247) were single (Table 2). A statistically significant association was observed between marital status and HPV positivity (χ2 [1] = 81.28, *p* < 0.001). Specifically, the rate of HPV-positive results was significantly higher among single individuals (56.3%) compared to married individuals (24.5%).

Respectively. However, single individuals had 3.96 times higher odds of testing positive compared to married individuals (95% CI [2.91, 5.39]), and the relative risk of a positive result was 2.29 times higher for singles (Table 2).

**Fig. 6:**
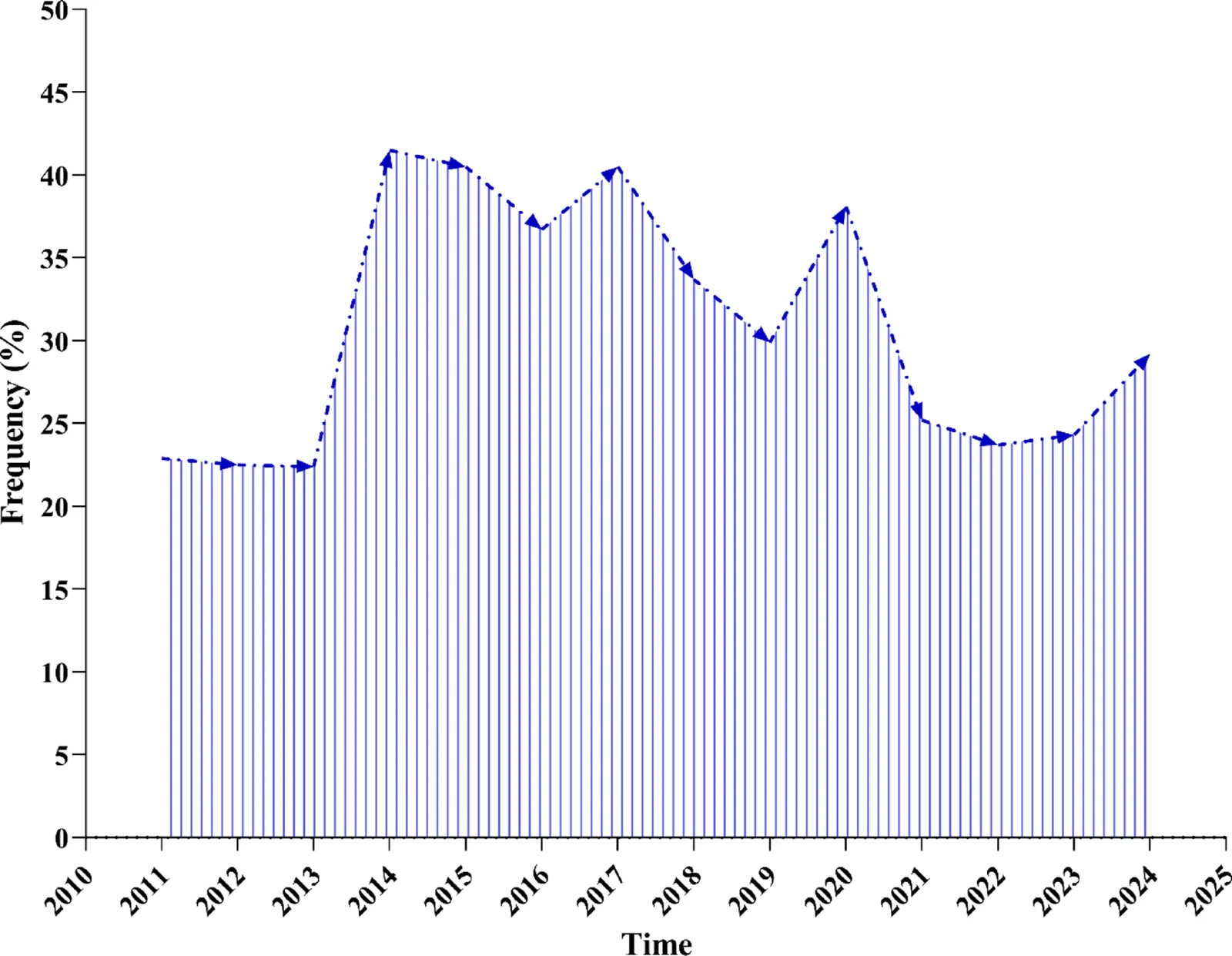
Annual trend of overall high-risk (HR) HPV infection rates from 2011 to 2024. The graph illustrates the fluctuations and trajectory of HR HPV positivity over the 14-year study period

Finally, the study examined the overall HR HPV infection rate from 2011 to 2024 (Fig. 6). The infection rate exhibited variability throughout this period. However, drawing definitive conclusions about trends in overall prevalence is challenging due to the evolving testing protocols and varying sensitivity of the diagnostic methods employed over the years.

## Discussion

Cervical cancer remains a significant global health concern, necessitating comprehensive strategies for prevention and control [28]. As highlighted by the WHO’s call to action in 2018, early screening of HPV, timely treatment, and expanded vaccination coverage are crucial for improving outcomes [29]. Determining the type-specific HPV prevalence is essential for formulating effective prevention strategies for cervical cancer [30]. This study offers a valuable examination of HPV genotype distribution from 2011 to 2024 about 6418 individuals in Tehran, using a precise molecular method. Our data indicates a prevalence of HPV approximately 42% within a referred population at a Saeed pathobiology and molecular lab, Tehran.

Prior research, such as a 2024 study by Hojjati et al., reported a 23% overall HPV prevalence among Iranian women, with rates varying widely from 2.2% to 97% across different regions [31]. Similarly, other studies.

in Tehran have reported HPV infection rates of 53% and 49.5% [32, 33]. These Iranian findings are generally consistent with prevalence rates observed in other populations. For example, a study of American women aged 18–59 reported a 39.9% prevalence of any HPV type [34]. These Iranian findings are broadly compatible with prevalence rates observed in international populations, where variations are attributed to lifestyle factors, sexual practices, financial limitations, awareness, and differences in the analytical sensitivity of the diagnostic assays used [35].

This study of 6418 individuals over a period of 14 years demonstrated age-related trends in HPV infection rates, with the highest prevalence in the 13–20 and 20–30 years age groups. While some studies in Iran have reported peak prevalence in the 30–40 years age group, our results align with other research indicating higher HPV prevalence among individuals under 30 years [3 36, 37]. The elevated infection rate in younger women is attributed to recent sexual activity and a less sensitized immune system, aligning with existing [2, 38]. The decreasing infection rates with age suggest viral clearance or potential reactivation during menopause, highlighting the importance of early HPV vaccination programs [39, 40]. A notable observation in our dataset is the disparity in HPV positivity rates between genders (40.8% in women vs. 63.3% in men). However, this difference must be interpreted with caution due to the distinct clinical indications for HPV testing in these populations. In our region, female HPV testing is predominantly driven by routine cervical cancer screening programs, capturing a large number of asymptomatic individuals. Conversely, male testing is rarely part of routine screening and is typically initiated due to symptomatic discomfort (e.g., genital warts) or contact tracing following a female partner’s abnormal diagnosis. Consequently, the male cohort represents a higher-risk, symptomatic, or exposed sub-population, naturally yielding a higher positive rate compared to the general female screening population. This difference in sample characteristics represents a limitation in making direct epidemiological comparisons between genders in our study. The increasing prevalence in Iran, influenced by factors like limited sex education and restricted vaccine access, underscores the urgent need for intervention [41, 42]. The cost of HPV vaccines, not covered by insurance, poses a barrier, necessitating effective national interventions and oversight to manage vaccine usage and curb rising infection rates.

The analysis of HPV genotypes in this study reveals a complex infection landscape characterized by a notable prevalence of co-infection with both low-risk (LR) and high-risk (HR) genotypes. While HR genotypes were more common overall (29.2%) compared to LR genotypes (24.8%), substantial 27.6% of individuals exhibited co-infection, suggesting potential synergistic effects. Gender-specific analysis showed a higher proportion of HR genotypes in males (36.7%) than females (28.6%). Although our findings show discrepancies when compared to other Iranian study [43], these differences in HPV prevalence among adjacent and relatively homogeneous regions are largely driven by sample inclusion criteria rather than regional heterogeneity alone. The positivity rate in universal health screening populations is inherently much lower than that seen in cohorts selected for clinical symptomatic testing or partner contact screening. Consequently, the composition of the enrolled population—specifically the balance between asymptomatic individuals undergoing routine examinations and symptomatic clinical patients—is the core factor causing prevalence bias across different epidemiological studies.

This study investigated the distribution of HPV genotypes in a population referred for HPV diagnosis, identifying 2718 infected cases and 34 distinct genotypes. The most frequent genotypes were 6, 16, 52, 31, 53, 66, 51, 11, 44, 54, 42, and 39. HPV-16 was the most prevalent high-risk (HR) genotype, while HPV-6 was the most common low-risk (LR) genotype. In this study, we found a lower prevalence of HPV-18 and a higher frequency of HPV-52 (3.6%), HPV-53 (3.1%), and HPV-31 (3.3%). These variations highlight the importance of investigating local epidemiological patterns for effective prevention and management of HPV-related diseases.

Single infections were the most common in this study (22.8%), followed by double infections (10.3%). Variations in infection patterns may be influenced by factors such as gender, sexual behavior, and geographic location, warranting further investigation to inform targeted prevention strategies. Regional and demographic factors must be considered when assessing HPV prevalence and associated risks. Multiple HPV infections are more common in men, potentially linked to a higher number of sexual partners [44].

This study, examining HPV history and clinical presentation in patients referred for HPV testing between 2023 and 2024, reveals important insights into HPV persistence and clearance. In this study, approximately 49.8% of patients who reported a prior positive HPV result tested positive again in the current assessment. However, a significant limitation of our study is the reliance on self-reported patient history for prior HPV status without access to previous laboratory records. Because we lack data on the specific genotypes of the initial infections and the exact time interval between the tests, we cannot definitively classify these current positive results as true persistent infections. It is entirely possible that some of these cases represent newly acquired infections with different HPV genotypes rather than the failure to clear the original virus. Future longitudinal studies with paired genotype data are required to accurately assess HPV persistence and clearance rates in this population.

Previous studies have identified several factors that are strongly associated with HPV acquisition, including marital status, lifetime number of sex partners, history of STIs, and oral contraceptive use [43, 45]. Notably, this earlier research demonstrated that having two or more lifetime sex partners and a history of STIs are the most significant determinants of high-risk HPV incidence [45]. A greater number of lifetime partners was associated with reduced clearance rates for any HPV [46]. These findings are contextualized by other studies. Allameh et al. (2022) reported an overall HPV clearance rate of 67%, with age, cervical histopathology, and parity as key risk factors for persistence [43]. A limitation of this study is the absence of data on the timing of initial infection and prior positive results, preventing a complete assessment of long-term clearance rates. Further research is needed to fully understand the dynamics of HPV infection and clearance, and to develop targeted prevention and treatment strategies.

The relationship between marital status and HPV infection rates presents a complex picture, warranting careful consideration of cultural and behavioral factors. While in our study revealed a significantly higher HPV prevalence among single individuals (56.3%) compared to their married counterparts (24.5%), another Iranian study reported similar rates in both groups (66.5% and 67%, respectively) [33]. This discrepancy might be hypothesized to relate to potential shifts in sexual behaviors, as single individuals may have a higher likelihood of engaging in multi-partner relationships; however, further targeted behavioral studies are required to confirm this. The absence of similar findings in older Iranian studies could be attributed to the social stigma surrounding such relationships, which likely discouraged the inclusion of marital status as a variable.

Most HPV infections are asymptomatic and result in no clinical disease. While some infections manifest as small genital warts on the vagina, penis, anus, or rarely the throat—which may be painful, itchy, bleed, or cause swollen glands the majority remain clinically silent [47]. This is supported by our findings, where 69.4% of HPV-positive individuals were entirely asymptomatic.

Furthermore, our specific analysis of clinical signs in women (Table 5) confirms that most general gynecological symptoms are poor predictors of HPV status. Conversely, 23.0% of HPV-negative individuals reported symptoms, suggesting alternative etiologies for their conditions. These findings align with research by Mao et al., who found no association between HPV infection and non-specific symptoms like discharge, itching, or burning [48]. Unlike genital warts, which are highly suggestive of low-risk HPV infection, high-risk genotypes often remain undetectable until advanced cytological changes occur. This convergence of evidence underscores the critical importance of routine molecular screening. Because women may harbor the virus without any specific clinical indicators, the lack of symptoms should not be a deterrent for HPV testing.

Variations in HPV detection methodologies significantly impact reported prevalence rates [49]. In this study, overall detection rates for INNO-LiPA, HybriSpot, cobas 4800, and Sansure Real-time PCR were 55.7%, 61.5%, 17.2%, and 52.1%, respectively. The pronounced discrepancy in positivity rates—most notably between HybriSpot (61.5%) and cobas 4800 (17.2%)—merits careful technical and epidemiological consideration. First, this variance is driven by distinct diagnostic targets; the cobas 4800 system is restricted to 14 h genotypes, whereas HybriSpot and INNO-LiPA capture a wide array of both HR and LR genotypes. Given the high prevalence of LR-HPV 6 in our cohort, assays capable of LR detection naturally exhibit higher overall positivity. While cobas 4800 offers high clinical specificity [50], HybriSpot’s multiplex PCR and DNA Flow Technology enhance analytical sensitivity across a broader genotype spectrum.

Second, differences in DNA extraction protocols directly impacted assay sensitivity. For the INNO-LiPA, HybriSpot, and Sansure assays, viral DNA was extracted using a standalone Roche extraction kit. As verified by routine NanoDrop quality control, this method yielded higher DNA concentrations compared to the automated, closed-system extraction of the cobas 4800, potentially improving the Limit of Detection (LOD) for low-viral-load infections. Finally, these diagnostic methods were utilized sequentially across different years, reflecting a shifting patient demographic. As depicted in Fig. 5A, patient referral volumes evolved over time, and Fig. 6 demonstrates that HR-HPV detection rates remained stable at approximately 30%–40% during the INNO-LiPA and HybriSpot periods. Therefore, the lower overall positivity observed in later years correlates primarily with evolving clinical practices rather than solely analytical differences. Early testing was predominantly diagnostic for symptomatic cases—especially those with genital warts, where HPV 6 is the predominant type—whereas later years saw a massive influx of routine, asymptomatic cervical cancer screening referrals. This shift toward a general screening population inevitably dilutes the overall observed prevalence. Further underscoring methodological effects, our comparison of LiPA and Sansure-24/26 assays showed 91.3% and 89.7% agreement across compatible genotypes, and literature indicates other assays like the Vitro HPV test show comparable clinical sensitivity to cobas for CIN2 + lesions [50]. Ultimately, these findings highlight that assay target range, extraction yields, and the specific clinical indications of the tested population are all critical determinants in interpreting longitudinal HPV prevalence data. Finally, the study examined the overall HR HPV infection rate from 2011 to 2024 (Fig. 6). The infection rate exhibited variability throughout this period. However, drawing definitive conclusions about trends in overall prevalence is challenging due to the evolving testing protocols and varying sensitivity of the diagnostic methods employed over the years. The increasing prevalence of HPV among sexually active Iranians, regardless of economic status, cultural habits, sampling periods, and lifestyles, marital status, or age, underscores the urgent need for intervention. This data can inform future research, influence public health policies, and contribute to the design of vaccines and national vaccination initiatives [51]. The rising rates of genital tract HPV infection in Iran, potentially linked to a lack of sex education and limited vaccine access, mirror trends observed globally. These trends highlight the impact of early sexual activity, multiple partners, and inadequate preventive measures on HPV infection rates.

This study, while providing valuable insights into HPV prevalence, is subject to several limitations. Notably, vaccination status for HPV was unavailable, a critical factor influencing test results. The study population, comprised of individuals seeking HPV testing at the laboratory, likely represents a higher-risk group with significant symptoms, thus limiting generalizability to the broader population. Furthermore, the absence of demographic data, such as occupation and number of sexual partners, restricts a more comprehensive analysis. The lack of cytology information for positive samples also prevents correlation between HPV genotypes and cytological findings. Potential errors in ThinPrep cytologic test results further contribute to the study’s limitations. Finally, the assumption of comparable infection prevalence throughout the 2011–2024 period may not fully account for the evolving impact of HPV vaccination on non-vaccine- type prevalence. While these limitations exist, this study provides valuable insight into HPV genotype distribution within the target population, informing future policy decisions.

## Conclusion

In conclusion, this study contributes valuable insights into HPV prevalence and genotype distribution in samples collected over a 14-year period in Tehran. The high prevalence of HPV infection, particularly with high-risk genotypes like HPV 16, 52, and 31, coupled with the highest prevalence observed in the 20–30 year age group and HPV-16 being common in younger ages, underscores the necessity for sustained vigilance and comprehensive cervical cancer prevention strategies. These strategies should encompass targeted education for younger populations, widespread vaccination programs, and consistent screening efforts. While the presented incidences are modeled estimates, the substantial burden of HPV highlights the importance of ongoing monitoring of HPV-associated diseases, including cancers, to inform and refine prevention measures. Future research should focus on genotype-specific risks and outcomes to enable more targeted interventions and ultimately safeguard women’s cervical health.

## Data Availability

The datasets generated and/or analyzed during the current study are available from the corresponding author on reasonable request. Due to the large volume of data collected over 14 years and the inclusion of clinical information, the datasets are not publicly available. All data have been fully anonymized to protect participant privacy.
